# Soil texture analysis revisited: Removal of organic matter matters more than ever

**DOI:** 10.1371/journal.pone.0178039

**Published:** 2017-05-18

**Authors:** Johannes Lund Jensen, Per Schjønning, Christopher W. Watts, Bent T. Christensen, Lars J. Munkholm

**Affiliations:** 1 Department of Agroecology, Aarhus University, Tjele, Denmark; 2 Department of Sustainable Agriculture Sciences, Rothamsted Research, Harpenden, United Kingdom; RMIT University, AUSTRALIA

## Abstract

Exact estimates of soil clay (<2 μm) and silt (2–20 μm) contents are crucial as these size fractions impact key soil functions, and as pedotransfer concepts based on clay and silt contents are becoming increasingly abundant. We examined the effect of removing soil organic matter (SOM) by H_2_O_2_ before soil dispersion and determination of clay and silt. Soil samples with gradients in SOM were retrieved from three long-term field experiments each with uniform soil mineralogy and texture. For soils with less than 2 g C 100 g^-1^ minerals, clay estimates were little affected by SOM. Above this threshold, underestimation of clay increased dramatically with increasing SOM content. Silt contents were systematically overestimated when SOM was not removed; no lower SOM threshold was found for silt, but the overestimation was more pronounced for finer textured soils. When exact estimates of soil particles <20 μm are needed, SOM should always be removed before soil dispersion.

## Introduction

Reliable estimates of clay- (<2 μm) and silt-sized (2–20 μm) particles in soil are now more important than ever as the use of pedotransfer functions are becoming increasingly abundant. Based on clay and silt contents, pedotransfer functions include attempts to predict soil water characteristics [1, 2], solute transport [3] and particle density [4]. Using reference values from conventional soil texture analysis, soil spectroscopy has been adopted as rapid methods to predict clay and silt contents [5–7]. Prediction of soil clay content from soil water characteristics is another rapidly progressing line of research based on pedotransfer concepts [8, 9].

For our ongoing research on the potential of soil clay/carbon and Fines20 (mineral particles <20 μm)/carbon ratios in defining critical low soil organic carbon (SOC) contents in agricultural soils [10, 11], it is essential to have access to exact values for clay and silt contents. This need is amplified by a recent study attempting to incorporate clay/SOC ratios to map the impact of management on soil quality at European scale [12].

Removal of soil organic matter (SOM) is recommended as a pretreatment before particle size analysis (e.g., [13]) to ensure effective dispersion of micro-aggregates. The internationally published studies originally underpinning the effect of SOM removal on estimates of clay and silt contents date back many decades [14–16]. These studies were based on a limited number of samples retrieved from contrasting sites, which prevent quantification of the effect of SOM *per se* on clay and silt estimates. Moreover, the historic studies applied less reliable methods for determination of SOC such as dichromate oxidation/titration and loss-on-ignition converted to SOC by division with the factor 1.724, a factor with a dubious scientific foundation [17]. Thus, we found it necessary to revisit this fundamental issue of soil texture analysis and examine in more detail the quantitative significance of SOM removal on clay and silt estimates.

This study quantifies the effect of SOM removal by H_2_O_2_ on the determination of clay and silt-sized particles using samples covering a wide range of SOC and clay contents. The samples were retrieved from three long-term field experiments each with a uniform mineralogical and textural composition.

## Materials and methods

To obtain soils with a gradient in SOC but with a uniform mineralogical and textural composition, samples were retrieved from three long-term field experiments in plots with contrasting management.

### Highfield ley-arable experiment

In the Highfield Experiment at Rothamsted Research, UK (51°80’N, 00°36’W), four treatments were sampled: BF, bare-fallow maintained free of vegetation since 1959; A, arable rotation with winter cereals since 1948; LA, ley-arable rotation with three-year grass/clover ley followed by three years arable since 1948; RG, grassland ploughed and reseeded to grass in 1948. The A, LA and RG treatments were embedded in a randomized block design with four field replicates, whereas the four BF plots were not part of the original design and are located at one end of the experiment. The soil is a silt loam soil belonging to the Batcombe series ([18]; Chromic Luvisol (WRB) or Aquic Paleudalf (USDA Soil Taxonomy System)). Details of the experiment are given in the electronic Rothamsted Archive (www.era.rothamsted.ac.uk). Sample request and transfer of Highfield soils were issued by the Farm and Field Experiment Committee (FFEC) at Rothamsted Research.

In spring of 2015, bulk soil (6–15 cm depth) was sampled at three positions within each of four replicate plots providing a total of 48 samples (4 treatments x 4 replicates x 3 sampling positions).

### Bad Lauchstädt static fertilizer experiment

We revisited previously published data on soil texture and SOC for the Bad Lauchstädt long-term static fertilizer experiment, Germany (51°24’N, 11°23’E). Bulk soil (2–15 cm depths) was sampled in spring 2008 from six different fertilization treatments in a field grown with a 4-year crop rotation (winter wheat (*Triticum aestivum*), sugar beet (*Beta vulgaris*), spring barley (*Hordeum vulgare*), and potato (*Solanum tuberosum*)) [19]. Animal manure (AM) was applied every 2 years in rates of 0, 20 or 30 Mg ha^-1^. Half of the plots addressed received no additional fertilizer, while the other half was dressed with nitrogen, phosphorus and potassium depending on the nutrients in the applied AM. There were no field replicates in the experiment. The experiment was established in 1902 on a silt loam soil and is classified as a Haplic Chernozem (WRB). More details are given in [19].

### Askov animal manure and mineral fertilizers experiment

Previously published data on soil texture and SOC for the Askov long-term experiment on animal manure and mineral fertilizers, Denmark (55°28’N, 09°07’E) was revisited. Bulk soil (6–15 cm depth) was sampled in autumn 2014 from four different fertilization treatments in a field grown with a 4-year crop rotation (winter wheat (*Triticum aestivum*), silage maize (*Zea mays*), and spring barley (*Hordeum vulgare*) undersown with a grass-clover mixture that is used for cutting in the subsequent production year) [20]. The nutrient treatments were: unfertilized; ½ mineral fertilizer (initiated in 1923); 1 mineral fertilizer; 1½ animal manure. The treatments were embedded in a block design with three replicates providing a total of 12 samples. The experiment was established in 1894 on a sandy loam soil and is classified as an Aric Haplic Luvisol (WRB) and Ultic Hapludalf (USDA Soil Taxonomy System). More details are given in [20].

### Clay, silt and soil organic carbon

Contents of clay (<2 μm) and silt (2–20 μm) was determined on air dry bulk soil (< 2 mm) by the hydrometer method [13] using the ASTM 152H hydrometer. First, the soils were tested for CaCO_3_ by adding a few droplets of 10% HCl, but none was found. Then one subsample (50 g soil) was treated with 35% H_2_O_2_ in an acidic solution under heating to remove SOM while another subsample was left untreated. After removal of H_2_O_2_ by boiling, the sample was washed with demineralized water until pH 6. Subsequently, the two sets of subsamples were dispersed by the same procedure. All lab work took place at 20°C. Each subsample was placed in 500-mL plastic bottles and 50 mL of 0.08 mol L^-1^ sodium pyrophosphate (Na_4_P_2_O_7_) and 200 mL demineralized water were added and the solution shaken end-over-end for 18 h. After transfer to 1000-mL sedimentation glass cylinder, demineralized water was added until 1000 mL and hydrometer readings were taken after 6.5 and 120 min to determine the <20 μm fraction, and after 2 and 18 h to determine the <2 μm fraction. The SOC content was determined on separate ball-milled sub-samples using dry combustion (Flash 2000 NC Soil Analyzer, Thermo Fisher Scientific).

Contents of SOC, clay and silt are related to oven-dry weight (105°C for 24 h) of the SOM-free mineral fraction. Correction factors for particle density were applied in calculating clay and silt contents (Table 1 and 3 in [21]). Values for individual soil samples are shown in S1 Dataset.

### Statistics

Linear regressions and ANOVA were applied using the R-Project software package Version 3.1.1 (R Foundation for Statistical Computing). The broken-stick model was fitted using the *segmented* function and the significance of the change point was assessed using the *davies*.*test* implemented in the *segmented* package in R. A simple piece-wise linear model was used:

$$y=\beta_{0}+\beta_{1}\left(x\right)+\beta_{2}{\left(x-c\right)}^{+}+e$$ (1)

where *y* is the dependent variable, *x* is the independent variable, *c* is the change point and *e* are the residual error [22].

## Results and discussion

The soils at Highfield ranged from 0.80 to 4.27 g C 100 g^-1^ minerals, with the smallest SOC content under BF and the highest under RG. The soils at Bad Lauchstädt and Askov ranged from 1.63–2.57 and 0.87–1.41 g C 100 g^-1^ minerals, respectively, with the smallest SOC content under the unfertilized treatments and the highest under the treatments receiving the highest amount of animal manure. A stringent test of the effect of SOC on clay and silt estimates requires that differences between treatments in contents of clay and silt are insignificant. Because the BF treatment was located only at one end of the field experiment, it was tested whether clay and silt in pretreated soil samples differed between BF and the other treatments. For clay, no significant difference was found (Table 1), whereas silt contents differed with 1.6 g 100 g^-1^ minerals (6% less silt for the BF treatment). It was not possible to test for differences between treatments at Bad Lauchstädt due to the lack of replicates, but in general the texture of the plots were similar [19], although the clay and silt in pretreated soil samples were slightly correlated with treatments having the highest SOC content. Contents of clay and silt between treatments were insignificant at Askov [20].

**Table 1:** Average values (g 100 g^-1^ minerals) of clay, silt and Fines20 for hydrogen peroxide treated soils from Group 1 and Group 2 at Highfield. P-values for testing differences between the two groups are indicated and were calculated by a one-way ANOVA.

	Group 1^1)^	Group 2^2)^	*p-*value
**Clay (<2 μm)**	27.0	26.1	0.21
**Silt (2–20 μm)**	24.9	26.5	<0.001
**Fines20 (<20 μm)**	51.9	52.6	0.34

^1)^ Group 1 –Bare-fallow.

^2)^ Group 2 –Arable, Ley-Arable and Reseeded Grass.

**Fig 1:**
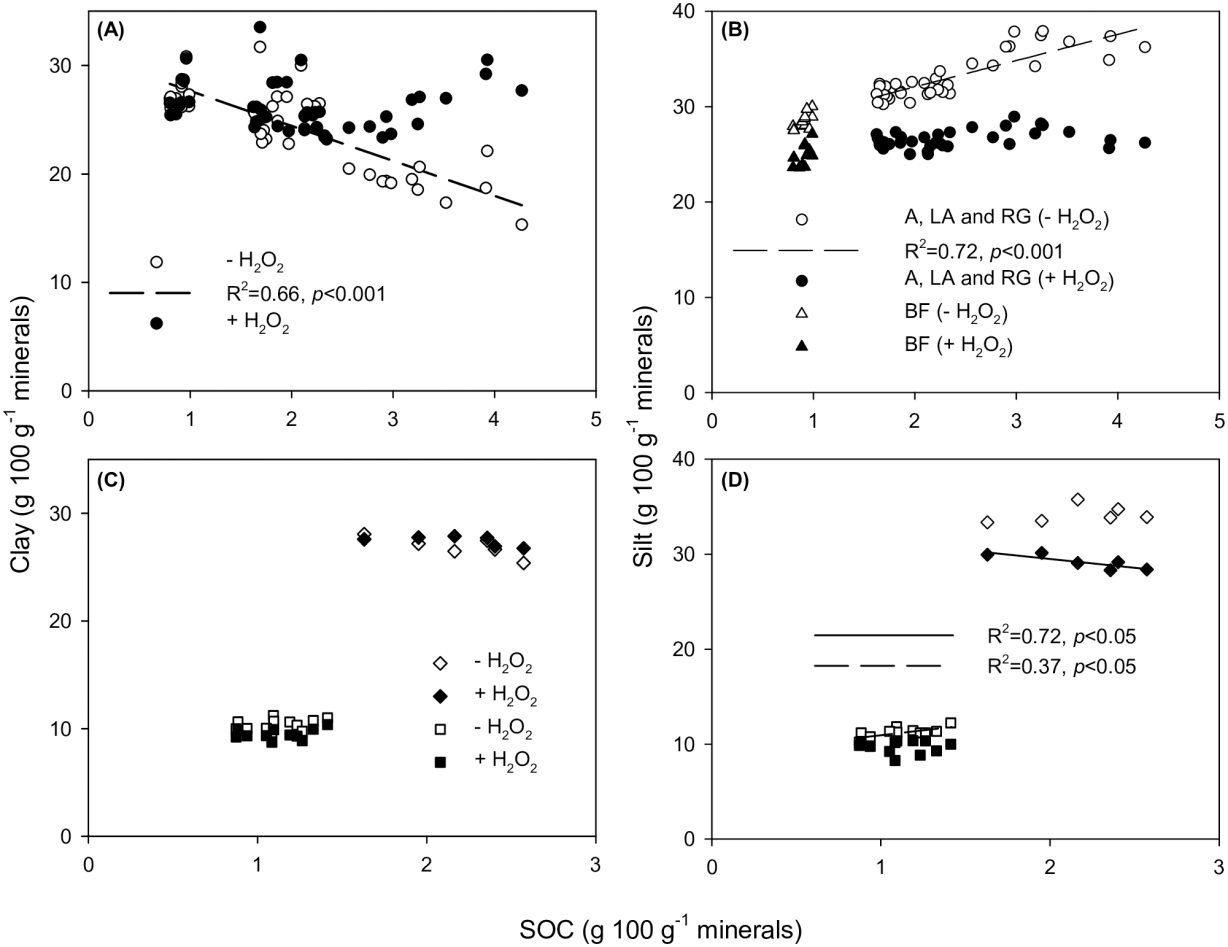
Clay (<2 μm) and silt (2–20 μm) content as a function of SOC content for soil samples pretreated with hydrogen peroxide and without pretreatment. (A) Clay content plotted against SOC content for soil samples pretreated with hydrogen peroxide (black symbols) and without pretreatment (white symbols) at Highfield. The linear regression line, R^2^ and *p*-value for non-pretreated soil samples are indicated (*n* = 48). (B) Silt content plotted against SOC content for soil samples pretreated with hydrogen peroxide and without pretreatment at Highfield. The linear regression line, R^2^ and *p*-value for non-pretreated arable (A), ley-arable (LA) and reseeded grass (RG) soil samples are indicated (white circles, *n* = 36). The bare-fallow (BF) soil samples are shown with triangle symbols (*n* = 24). (C) Clay content plotted against SOC content for soil samples pretreated with hydrogen peroxide and without pretreatment at Bad Lauchstädt (diamond symbols) and Askov (square symbols). (D) Silt content plotted against SOC content for soil samples pretreated with hydrogen peroxide and without pretreatment at Bad Lauchstädt and Askov. The linear regression line, R^2^ and *p*-value for pretreated and non-pretreated soil samples for Bad Lauchstädt (*n* = 6) and Askov (*n* = 12), respectively, are indicated.

There was a strong negative relationship between SOC and clay estimates for soils without SOM removal at Highfield (R^2^ = 0.66, *p*<0.001), whereas the relationship was non-significant for samples pretreated with H_2_O_2_ (R^2^ = 0.00, *p* = 0.84) (Fig 1A). Similarly, there was a strong positive relationship between SOC and silt estimates when SOM was not removed (R^2^ = 0.72, *p*<0.001) and no significant relationship for H_2_O_2_ treated samples (R^2^ = 0.11, *p* = 0.053) (Fig 1B). The linear regression analysis for silt excluded the BF soils due to the significant difference in silt content between BF and the other treatments. There was a non-significant negative relationship between SOC and clay estimates both without SOM removal (R^2^ = 0.61, *p* = 0.06) and for samples pretreated with H_2_O_2_ (R^2^ = 0.36, *p* = 0.20) at Bad Lauchstädt (Fig 1C). The SOM effect was close to significant and more pronounced for soils without SOM removal being in agreement with the results from Highfield. There was a non-significant relationship between SOC and silt estimates when SOM was not removed (R^2^ = 0.12, *p* = 0.52), whereas there was a negative relationship for H_2_O_2_ treated samples (R^2^ = 0.72, *p*<0.05) (Fig 1C). This is in line with the results from Highfield; since the silt content with SOM removal decreased slightly with an increase in SOC, a more or less unaffected silt content without SOM removal was expected. For Askov there was a non-significant relationship between SOC and clay estimates both when SOM was not removed (R^2^ = 0.09, *p* = 0.36) and for samples pretreated with H_2_O_2_ (R^2^ = 0.10, *p* = 0.31) (1C). The SOM effect on silt estimates was in agreement with results from Highfield and Bad Laucstädt with a positive relationship between SOC and silt estimates when SOM was not removed (R^2^ = 0.37, *p*<0.05) and a non-significant relationship for H_2_O_2_ treated samples (R^2^ = 0.00, *p* = 0.84) (Fig 1D). In general it can be seen, that the presence of SOM caused a systematic error in clay and silt estimates.

We suggest that the underestimation of clay and overestimation of silt with increasing SOC is due to incomplete dispersion of soil aggregates smaller than 20 μm. Silt-sized micro-aggregates made up of SOM-clay complexes will settle faster and be quantified as silt even though, if fully dispersed, they should be classified as clay. Alternatively, SOM and clay particles may flocculate after dispersion and be classified as silt although flocculation is less likely in a sodic solution with a low particle concentration [23].

**Fig 2:**
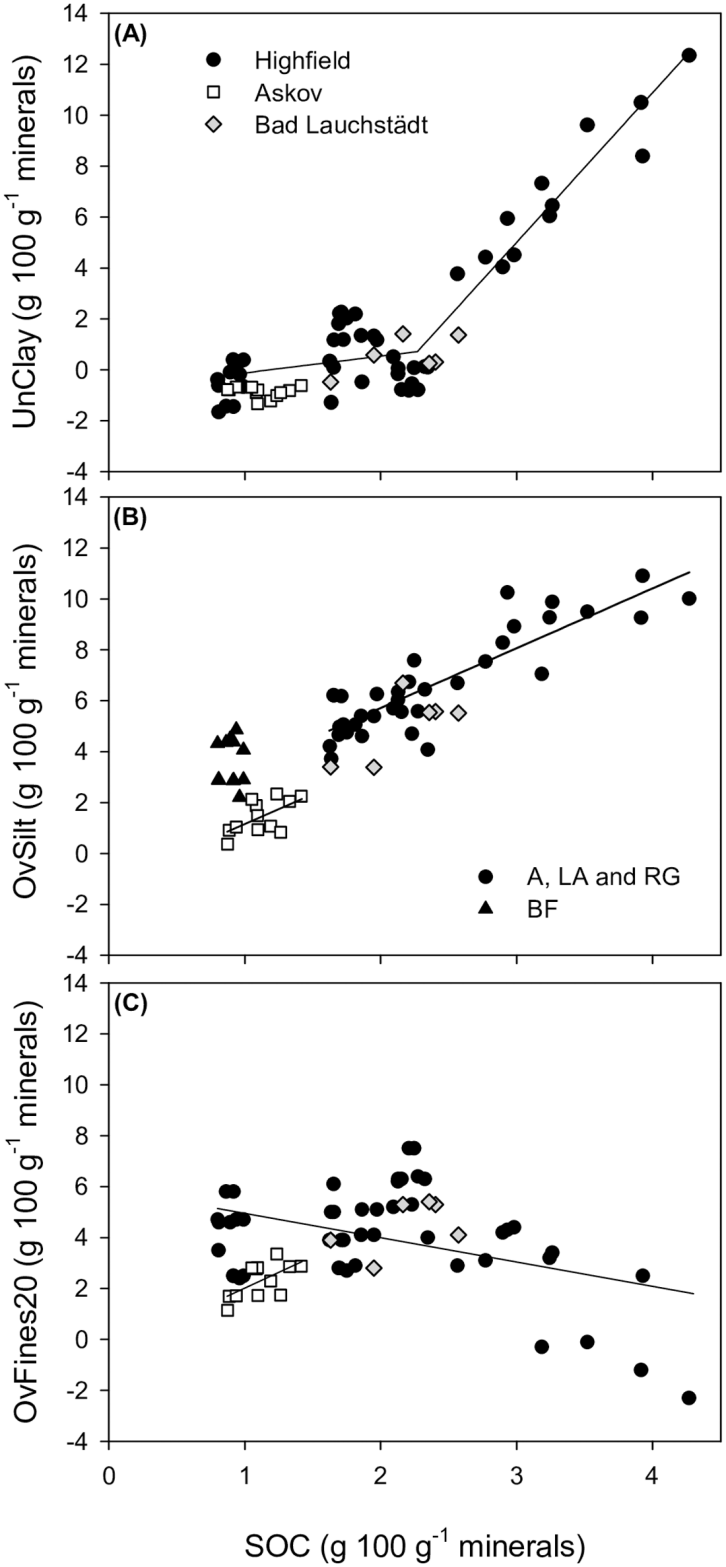
Underestimation of clay content (<2 μm), overestimation of silt content (2–20 μm) and overestimation of Fines20 content (<20 μm) caused by omitting hydrogen peroxide (H_2_O_2_) pretreatment as a function of SOC content. (A) Underestimation of clay content (UnClay) caused by omitting H_2_O_2_ pretreatment plotted against SOC content. The soil samples from Highfield, Bad Lauchstädt and Askov are shown with black, grey and white symbols, respectively. The broken-stick model is indicated for Highfield (*n* = 48). (B) Overestimation of silt content (OvSilt) caused by omitting H_2_O_2_ pretreatment plotted against SOC content. The linear regression line for Askov (*n* = 12) and for the Highfield arable (A), ley-arable (LA) and reseeded grass (RG) soil samples are indicated (circle symbols, *n* = 36). The bare-fallow (BF) soil samples are shown with triangle symbols (*n* = 12). (C) Overestimation of Fines20 content (OvFines20) caused by omitting H_2_O_2_ pretreatment plotted against SOC content. The linear regression lines for Askov (*n* = 12) and Highfield (*n* = 48) are indicated.

The underestimation of clay caused by omitting the H_2_O_2_ treatment increased with increasing SOC content (Fig 2A). To establish the SOC content below which SOM removal becomes unnecessary, a broken-stick model was fitted to the Highfield data. This threshold value for clay was 2.27 g C 100 g^-1^ minerals (95% confidence interval, 2.03–2.52 g C 100 g^-1^ minerals). This point of change was highly significant (*p*<0.001), and the following piecewise linear regression equation can be used to model the underestimation of clay (UnClay) at Highfield:

$$\text{UnClay}=-0.78\;\left(p=0.19\right)+0.66\left(p=0.07\right)\text{SOC}+5.22\left(p<0.001\right){\left(\text{SOC}-2.27\right)}^{+},\;{\text{R}}^{2}=0.90$$ (2)

The last term in the equation is only applicable for SOC contents above 2.27 g C 100 g^-1^ minerals. No threshold was observed for Bad Lauchstädt (*p* = 0.75) and Askov (*p* = 0.10), and the linear relationship between UnClay and SOC was non-significant (Bad Lauchstädt; R^2^ = 0.37, *p* = 0.19 and Askov; R^2^ = 0.01, *p* = 0.77). The overestimation of silt increased with an increase in SOC (Fig 2B) and was linearly related to SOC at Highfield and Askov and close to significant at Bad Lauchstädt (R^2^ = 0.52, *p* = 0.11). No threshold was observed at Highfield (*p* = 0.62), Askov (*p* = 0.70) and Bad Lauchstädt (*p* = 0.61). The following linear regression can be used to model the overestimation of silt (OvSilt) at Highfield:

$$\text{OvSilt}=1.03\left(p<0.001\right)+2.35\left(p<0.001\right)\text{SOC},{\text{ R}}^{2}=0.76$$ (3)

And the following linear regression can be used to model OvSilt at Askov:

$$\text{OvSilt}=-1.19\left(p=0.30\right)+2.35\left(p<0.05\right)\text{SOC},{\text{ R}}^{2}=0.37$$ (4)

The models show that SOM removal is critical for estimating clay in soils with more than 2 g C 100 g^-1^ minerals, whereas an unbiased estimate of silt requires SOM removal regardless of SOC content. For example, with no SOM removal, clay will be underestimated by 19% and silt overestimated by 30% in a soil with 3 g C 100 g^-1^ minerals (using Eqs 2 and 3; based on Highfield data). The data from Bad Lauchstädt and Askov are in general agreement with the broken-stick model on Highfield (Fig 2A), whereas the OvSilt for Askov is lower (Fig 2B). Interestingly, the slope estimate for Eqs 3 and 4 is identical indicating a similar effect of SOM on OvSilt between the two sites. The lower OvSilt for Askov compared with Highfield and Bad Lauchstädt may be due to textural differences with Askov being coarser textured.

Standard protocols for estimation of clay- and silt-sized particles are the hydrometer and pipette methods, both based on gravitational sedimentation following soil dispersion [13]. We relied on the hydrometer approach. The results obtained for clay and silt in H_2_O_2_ treated soils from Highfield are consistent with previous estimates based on the pipette method [18].

Most protocols propose the use of 30% H_2_O_2_ for SOM removal before soil dispersion. We recognize that the prescribed H_2_O_2_ treatment does not remove all SOM from the soil. Typically, 80 to 90% of the initial SOC content is removed by the prescribed protocol (e.g., [24–26]). We also recognize that the H_2_O_2_ treatment may dissolve mineral constituents including vermiculite, mica and smectite in particular [27]. The clay fraction of Highfield and Bad Lauchstädt soil do have a higher content of smectite and vermiculite than Askov [28–30], which may explain the slightly higher loss of Fines20 for Highfield and Bad Lauchstädt than Askov (Fig 2C).

We also acknowledge that our study was based on a coarse sandy soil and two silt loams each having different clay mineralogies. However each of the soils considered here encompassed a reasonably wide range of SOC contents resulting from contrasting long-term agricultural treatments. We advocate similar studies based on long-term field experiments located on other soil types to examine any additional effects of differences in soil textural composition and mineralogy. Studies on soils dominated by low-activity clays such as kaolinite and Fe and Al oxide minerals are in particular needed.

## Conclusions

The presence of SOM induced systematic errors in the estimation of clay and silt contents. For soil with less than 2 g C 100 g^-1^ minerals, clay estimates were little affected by SOM. An overestimation of silt occurred at all SOC contents considered here. The overestimation of the silt fraction was greater for the silt loams compared to the sandy loam. Consequently, SOM should always be removed before soil dispersion when exact estimates of particles <20 μm are needed.

## Supporting information

S1 Dataset Data used in Fig 1, Table 1 and for developing the models in Fig 2. The soil characteristics are expressed in relation to oven-dry weight of the SOM-free mineral fraction.
(PDF)
