# Supplementary material for: Soil texture analysis revisited: Removal of organic matter matters more than ever
Source: PLoS One. 2017 May 18;12(5):e0178039. doi: 10.1371/journal.pone.0178039 (PMC5436882; doi:10.1371/journal.pone.0178039)
Supplement: S1 Dataset — The soil characteristics are expressed in relation to oven-dry weight of the SOM-free mineral fraction. (PDF) [file pone.0178039.s001.pdf]

S1 Dataset. Data used in Fig 1, Table 1 and for developing the models in Fig 2.

The soil characteristics are expressed in relation to oven-dry weight of the SOM-free mineral fraction.

Site	Sample ID	Treatment[1)]	Clay (< 2 µm) (- H_2O_2)	Clay (< 2 µm) (+ H_2O_2)	Silt (2-20 µm) (- H_2O_2)	Silt (2-20 µm) (+ H_2O_2)	Fines20 (<20 µm) (- H_2O_2)	Fines20 (<20 µm) (+ H_2O_2)	SOC
			(g 100 g^{-1} minerals)	(g 100 g^{-1} minerals)	(g 100 g^{-1} minerals)	(g 100 g^{-1} minerals)	(g 100 g^{-1} minerals)	(g 100 g^{-1} minerals)	(g 100 g^{-1} minerals)
Highfield	3-71	BF	26.9	25.5	28.0	23.6	54.9	49.1	0.86
	3-84	BF	28.0	26.6	28.1	23.7	56.1	50.3	0.91
	3-115	BF	26.9	26.5	27.9	23.6	54.9	50.2	0.80
	4-69	BF	26.4	26.3	28.4	23.9	54.8	50.2	0.89
	4-70	BF	27.1	25.4	27.6	24.6	54.7	50.1	0.81
	4-76	BF	26.0	25.4	27.5	24.6	53.6	50.1	0.81
	7-1	BF	27.3	26.6	28.9	24.9	56.2	51.5	0.99
	7-2	BF	26.2	26.6	30.0	27.1	56.3	53.8	0.99
	7-3	BF	26.2	26.6	28.8	25.9	54.9	52.5	0.91
	8-8	BF	28.3	28.7	28.9	26.1	57.3	54.8	0.92
	8-38	BF	30.8	30.6	27.6	25.4	58.5	56.1	0.96
	8-94	BF	28.6	28.7	29.8	24.9	58.3	53.6	0.94
	10-58	RG	15.3	27.7	36.2	26.2	51.6	53.9	4.27
	10-78	RG	18.7	29.2	34.9	25.6	53.6	54.8	3.91
	10-95	RG	22.1	30.5	37.4	26.5	59.5	57.0	3.93
	14-8	A	23.2	25.3	30.8	26.1	54.0	51.3	1.75
	14-42	A	24.0	25.2	31.1	26.0	55.1	51.2	1.72
	14-84	A	22.9	25.1	32.1	25.9	55.0	51.1	1.71
	17-23	RG	19.9	24.4	34.3	26.8	54.3	51.1	2.77
	17-61	RG	19.3	25.3	36.3	26.1	55.7	51.3	2.93
	17-102	RG	18.5	24.6	37.5	28.2	56.0	52.8	3.24
	20-1	A	23.7	25.9	31.2	26.3	54.9	52.2	1.70
	20-43	A	24.7	24.8	32.4	26.2	57.1	51.0	1.65
	20-143	A	25.0	26.2	32.1	25.9	57.1	52.1	1.65
	24-107	A	31.7	33.5	30.2	25.6	62.0	59.1	1.69
	24-123	A	27.1	28.5	31.6	26.2	58.7	54.6	1.86
	24-132	A	26.2	28.4	32.4	27.3	58.6	55.7	1.81
	26-49	RG	19.3	23.4	36.3	28.0	55.6	51.3	2.90
	26-51	RG	20.5	24.2	34.5	27.8	55.0	52.1	2.56
	26-127	RG	19.2	23.7	37.9	28.9	57.0	52.6	2.98
	30-49	RG	19.5	26.8	34.2	27.2	53.7	54.0	3.18
	30-63	RG	17.4	27.0	36.8	27.3	54.2	54.3	3.52
	30-65	RG	20.6	27.1	37.9	28.0	58.6	55.1	3.26
	33-31	A	22.8	24.0	32.6	26.3	55.4	50.3	1.97

	33-65	A	25.8	26.2	31.3	27.1	57.2	53.3	1.62
	33-70	A	25.6	24.3	30.4	26.7	56.0	51.0	1.63
	11/12-1	LA	25.3	25.4	31.4	25.0	56.7	50.4	2.13
	11/12-2	LA	23.2	23.3	31.4	27.3	54.6	50.6	2.35
	11/12-3	LA	27.1	28.4	30.4	25.0	57.5	53.4	1.95
	15/16-1	LA	23.4	23.5	32.3	25.8	55.7	49.4	2.32
	15/16-2	LA	26.5	25.7	31.5	25.9	58.0	51.6	2.27
	15/16-3	LA	26.2	25.4	32.9	26.2	59.2	51.6	2.21
	25/26-1	LA	26.2	25.7	31.8	27.1	58.0	52.8	2.23
	25/26-2	LA	24.2	24.3	33.7	26.1	58.0	50.4	2.25
	25/26-3	LA	30.0	30.5	32.5	26.8	62.5	57.3	2.09
	45/46-1	LA	24.2	24.0	31.3	25.3	55.5	49.3	2.13
	45/46-2	LA	24.9	24.4	31.4	26.8	56.3	51.2	1.86
	45/46-3	LA	26.4	25.7	31.5	25.9	57.9	51.6	2.15
Bad Lauchstädt[2)]	-	M3+	25.4	26.8	33.9	28.4	59.3	55.2	2.57
	-	M3-	26.6	26.9	34.8	29.2	61.4	56.1	2.40
	-	M2+	27.5	27.7	33.9	28.3	61.4	56.0	2.36
	-	M2-	26.5	27.9	35.8	29.1	62.3	57.0	2.16
	-	M0+	27.2	27.8	33.5	30.1	60.7	57.9	1.95
	-	M0-	28.1	27.6	33.4	30.0	61.5	57.6	1.63
Askov[3)]	511	½NPK	10.6	9.4	11.4	10.4	22.1	19.8	1.19
	512	1NPK	10.3	9.3	11.2	8.9	21.5	18.2	1.23
	513	UNF	9.6	8.7	10.2	8.3	19.8	17.0	1.08
	524	1½AM	10.8	10.0	11.4	9.3	22.1	19.3	1.33
	525	½NPK	11.2	9.9	11.8	10.3	23.1	20.2	1.09
	526	UNF	10.6	9.9	11.2	10.3	21.8	20.1	0.88
	532	1½AM	11.0	10.4	12.2	10.0	23.3	20.4	1.41
	535	1NPK	10.7	9.9	11.3	10.3	22.0	20.2	1.09
	543	½NPK	10.0	9.4	10.8	9.8	20.8	19.1	0.94
	544	UNF	10.0	9.2	10.2	9.9	20.2	19.1	0.87
	553	1NPK	10.0	9.4	11.4	9.2	21.4	18.6	1.05
	555	1½AM	9.8	8.9	11.2	10.4	21.0	19.2	1.26

[1)] A - Arable; BF - Bare-fallow; LA - Ley-arable; RG - Reseeded grass.

M3+ - 30 t animal manure ha^{-1} 2 y^{-1} and mineral fertilizer; M3- - 30 t animal manure ha^{-1} 2 y^{-1}; M2+ - 20 t animal manure ha^{-1} 2 y^{-1} and mineral fertilizer; M2- - 20 t animal manure ha-1 2 y-1; M0+ - mineral fertilizer; M0- - unfertilized.

UNF - unfertilized; ½NPK - ½ mineral fertilizer; 1NPK - 1 mineral fertilizer; 1½AM - 1½ animal manure.

[2)] Data from Eden *et al.* (2012). Linking soil physical parameters along a density gradient in a loess-soil long-term experiment. Soil Sci.; 177: 1-11. [19] in manuscript.

[3)] Data from Jensen *et al.* (2017). Suboptimal fertilisation compromises soil physical properties of a hard-setting sandy loam. Soil Res. [20] in manuscript.
